# Pharmacokinetics of Caffeic Acid, Ferulic Acid, Formononetin, Cryptotanshinone, and Tanshinone IIA after Oral Administration of Naoxintong Capsule in Rat by HPLC-MS/MS

**DOI:** 10.1155/2017/9057238

**Published:** 2017-07-05

**Authors:** Jin Li, Yang Bai, Yun Bai, Ruichao Zhu, Wei Liu, Jun Cao, Mingrui An, Zhijing Tan, Yan-xu Chang

**Affiliations:** ^1^Tianjin State Key Laboratory of Modern Chinese Medicine, Tianjin University of Traditional Chinese Medicine, Tianjin 300193, China; ^2^Tianjin Key Laboratory of Phytochemistry and Pharmaceutical Analysis, Tianjin University of Traditional Chinese Medicine, Tianjin 300193, China; ^3^College of Material Chemistry and Chemical Engineering, Hangzhou Normal University, Hangzhou 310036, China; ^4^Department of Surgery, University of Michigan, Ann Arbor, MI 48109, USA

## Abstract

Naoxintong capsule (NXTC) was a famous patent medicine of Traditional Chinese Medicine (TCM) to treat cerebrovascular diseases in China. An LC-MS/MS method was developed for simultaneous determination of 11 major ingredients (paeoniflorin, ecdysterone, amygdalin, mulberroside A, caffeic acid, ferulic acid, salvianolic acid B, astragaloside IV, formononetin, cryptotanshinone, and tanshinone IIA) in NXTC in rat plasma. All analytes were separated on an Eclipse plus C_18_ column using a gradient mobile phase system of acetonitrile-0.1% formic acid aqueous solution. The lower limits of quantification of 11 ingredients were between 0.075 and 10 ng mL^−1^. The precision was less than 15% and the accuracies were between 85% and 115%. The results showed that caffeic acid, ferulic acid, formononetin, cryptotanshinone, and tanshinone IIA could be detected after oral administration of NXTC. The validated method was successfully applied to pharmacokinetic study of the caffeic acid, ferulic acid, formononetin, cryptotanshinone, and tanshinone IIA in rats after oral administration of NXTC at single and triple dose.

## 1. Introduction

Traditional Chinese Medicine (TCM) prescriptions have played an important role in prevention and treatment of diseases for thousand years in some Asia countries such as China, Japan, and Korea. Several TCMs were usually combined together as a prescription which is the main form of TCMs to improve the therapeutic efficacy and reduce the possible adverse effect through synergistic action in clinic. Most of the TCM prescriptions were applied to treating some diseases from the ancient times and their therapeutic effects have been demonstrated and proved by broad clinical practice. Considering the effectiveness of the TCM prescriptions, it was necessary to perform more in-depth science research for clarifying material base of TCM prescriptions.

Buyanghuanwutang, one of the famous TCM prescriptions from the ancient medical book* Yi Lin Gai Cuo* written by Qingren Wang in Qing Dynasty, mainly records the theory “promoting blood circulation and removing blood stasis.” Naoxintong capsule (NXTC) is a patent medicine designed on the base of Buyanghuanwutang. It has been widely used in China to treat cerebrovascular disease caused by deficiency of Qi and blood stasis such as hemiplegia and stroke. NXTC contains 11 plant medicines (*Astragali Radix* (Huangqi),* Paeoniae Radix Rubra* (Chishao),* Salviae miltiorrhizae Radix et Rhizoma* (Danshen), Persicae Semen (Taoren),* Angelicae Sinensis Radix* (Danggui),* Achyranthis bidentatae Radix* (Niuxi),* Chuanxiong Rhizoma* (Chuanxiong),* Spatholobi Stem* (Jixueteng),* Cinnamomi Ramulus* (Guizhi),* Carthami Flos* (Honghua) and* Mori Ramulus* (Sangzhi)), two resins (*Olibanum *(Ruxiang),* Myrrha *(Moyao)), and three animal medicines (*Scorpio *(Quanxie),* Pheretima* (Dilong), and* Hirudo* (Shuizhi)) [1]. As NXTC is a widely used patent medicine in clinic, there were many reports on modern pharmacological studies and clinical analysis. NXTC could protect brain tissue from ischemia-reperfusion injury through some different ways [2–5]. It also could cure cognitive dysfunctions and treat patients with vascular dementia and coronary heart disease [6, 7]. NXTC has beneficial effects on atherosclerosis treatment by reducing expression of iNOS mRNA and the NO level in the vessel wall [8]. It was demonstrated that NXTC protected H9c2 cardiomyoblasts from H_2_O_2_-induced oxidative injury by increasing antioxidant abilities, activating ERK_1/2_, and blocking Ca_2_^+^-dependent and mitochondria-mediated apoptosis [9]. NXTC protected against atherosclerosis through lipid-lowering and inhibiting dendritic cells maturation in mice model of atherosclerosis [10]. It could reduce the infarct size of acute myocardial infarction patients and promote the cerebral blood circulation and renovate cerebral damage in treating hypertensive cerebral hemorrhage after surgical management [11]. Furthermore, it was reported that NXTC can inhibit the development of diabetic retinopathy [12]. NXTC could obviously increase the effect on the catalytic activities of drug metabolising CYP2C19 enzyme [13]. All of these reports indicated that NXTC had many important pharmacological functions and excellent efficacy.

Pharmacokinetics of TCM could provide scientific evidence such as absorption, distribution, metabolism, excretion, and metabolism of chemical components contained in TCMs. It was well-known that chemical components were only absorbed into blood, reach a certain blood concentration, and exert pharmacological effects. Therefore, it was very useful to do pharmacokinetic study of multiple compounds of NXTC for exploring the active compounds. Considering its complicated compounds of TCM, it was needed to establish a suitable method for clarifying the relation of complicated compounds and clinical effect.

Ultrahigh-performance liquid chromatography (UHPLC) and HPLC-ESI/TOF have been published for quality control of NXTC [14, 15]. The characteristics of absorption of four components (ferulic acid, paeoniflorin, salvianolic acid B, and hydroxysafflor yellow A) from NXTC in intestines were studied by UHPLC methods [16]. However, none of them is involved in pharmacokinetic study of multiple compounds of NXTC among all methods mentioned above. To our knowledge, there were no pharmacokinetic studies about NXTC as a powerful analytical technique. Liquid chromatography-tandem mass spectrometry (LC-MS/MS) method has gained much attention in bioanalytical analysis [17, 18], since it owned the advantages such as excellent specificity and sensitivity. The eleven typical compounds including paeoniflorin, ecdysterone, amygdalin, mulberroside A, caffeic acid, ferulic acid, salvianolic acid B, astragaloside IV, formononetin, cryptotanshinone, and tanshinone IIA are usually selected as mainly ingredients of some TCM material prescribed in Chinese Pharmacopoeia. In our study, these 11 typical compounds were selected as markers. A specific and sensitive LC-MS/MS method was established and validated to simultaneously determine concentration of 11 compounds in rat plasma. The new method has been validated and successfully applied to the pharmacokinetic study of 5 typical compounds after oral administration of NXTC.

## 2. Experimental

### 2.1. Material and Reagents

Methanol (Tianjin Concord Science Co. Ltd., Tianjin, China) and acetonitrile (Dikma Technologies Inc., USA) were of HPLC grade. Formic acid was purchased from Sigma-Aldrich (St. Louis, MO, USA). Deionized water was prepared with a Milli-Q Academic ultrapure water system (Millipore, Milford, MA, USA). All other reagents were of analytical grade (Tianjin Concord Science Co. Ltd., Tianjin, China). Standard references including paeoniflorin, ecdysterone, amygdalin, mulberroside A, caffeic acid, ferulic acid, salvianolic acid B, astragaloside IV, formononetin, cryptotanshinone, tanshinone IIA, chlorogenic acid, geniposide, sennoside B, and puerarin were purchased from National Institute for the Control of Pharmaceutical and Biological Products (Beijing, China) and Must Bio-Technology Co., Ltd. (Chengdu, China). The NXTC was purchased from Shaanxi Buchang Pharmaceutical Co., Ltd. (Xi -an, China).

### 2.2. Preparation of Stock Solution, Calibration Samples, and Quality Control (QC) Samples

Stock solutions of paeoniflorin, ecdysterone, amygdalin, mulberroside A, caffeic acid, ferulic acid, salvianolic acid B, astragaloside IV, formononetin, cryptotanshinone, and tanshinone IIA were prepared separately in methanol to achieve a high concentration of 1.0 mg mL^−1^ and chlorogenic acid, geniposide, sennoside B, and puerarin were prepared as internal standards (ISs) with the same way to achieve a concentration of 100 ng mL^−1^. Appropriate volumes of the 11 standard solutions were mixed to obtain the working standard solution. All solutions were stored at 4°C.

A series of different concentrations of working solution were obtained by mixed appropriate volume of the standard solutions and diluting with methanol. Then calibration work solutions were prepared by adding 10 *μ*L of the series work standard solutions, 10 *μ*L IS and 10 *μ*L formic acid into 100 *μ*L blank rat plasma. The final concentrations of the series analytes were prepared ranging within 2.5–750 ng mL^−1^ for paeoniflorin, 5–1500 ng mL^−1^ for ecdysterone, 10–3000 ng mL^−1^ for amygdalin, salvianolic acid B, and astragaloside IV, 3–3000 ng mL^−1^ for mulberroside A and caffeic acid, 1.5–1500 ng mL^−1^ for ferulic acid, 0.075–75 ng mL^−1^ for formononetin and cryptotanshinone, 0.3–300 ng mL^−1^ for tanshinone IIA, respectively.

Quality control (QC) samples were prepared following the same sample preparation method described above at the concentrations of 7.5, 75, and 750 ng mL^−1^ for paeoniflorin, 15, 150, and 1500 ng mL^−1^ for ecdysterone and ferulic acid, 30, 300, and 3000 ng mL^−1^ for amygdalin, salvianolic acid B, mulberroside A, caffeic acid, and astragaloside IV, 0.75, 7.5, and 75 ng mL^−1^ for formononetin, cryptotanshinone, and 3, 30, and 300 ng mL^−1^ for tanshinone IIA, respectively. Calibration work solutions and QC samples were stored at 4°C until LC-MS/MS analysis.

### 2.3. LC-MS/MS Analysis

The LC-MS/MS analysis was carried out on an Agilent 1200 system (Agilent Corporation, USA) coupled with an API 3200 triple quadrupole mass spectrometer with a Turbo Ion Spray Ionization (ESI) source (Concord, Ontario, Canada). The Agilent 1200 system consisted of a vacuum degasser (G1322A), a binary pump (G1312A), and a Hip-ALS autosampler (G13678). Chromatographic separation was performed on an Eclipse plus C18 column (4.6 × 100 mm, 1.8 *μ*m) with a security guard C_18_ column (2.1 mm × 12.5 mm, 5 *μ*m) (Agilent, USA). The mobile phases consisted of acetonitrile (A) and formic acid aqueous solution (B). The linear gradient was 30% A-30% A (0–10 min); 30%–80% A (10–15 min); 80%–95% A (15–20 min); 95%-95% A (20–30 min). Reequilibration time after gradient elution was 5 min. The column temperature was maintained at 25°C and the flow rate was set at 0.300 mL min^−1^. ESI-MS spectra were acquired in both negative and positive ion multiple reaction monitoring (MRM) mode. The conditions of source and MRM parameters were shown in Table 1. Data collection and peak calculations were performed by Analyst 1.4.2 software (AB MDS Sciex).

### 2.4. Preparation of Sample

The ISs solution (10 *μ*L) and formic acid (10 *μ*L) were added to plasma samples (100 *μ*L). Acetonitrile (370 *μ*L) was used to precipitate protein. The mixed solution was swirled for 1 min and then centrifuged at 14000 rpm for 10 min. The supernatant fluid was collected and then evaporated to dryness with a stream of N_2_ gas in a 40°C water bath. The residue was reconstituted in 100 *μ*L methanol, swirled for 1 min. The sample was centrifuged at 14000 rpm for 10 min. 10 *μ*L of the supernatant was injected into the LC-MS/MS system for analysis.

### 2.5. Method Validation

The method was validated in terms of specificity, lower limit of quantification (LLOQ), accuracy and precision, extraction recovery, matrix effect, and stability following the guidelines for bioanalytical method validation issued by the FDA Center for Drug Evaluation and Research.

#### 2.5.1. Specificity

The specificity of the method was investigated by comparing chromatograms of blank plasmas obtained from six different rats, with those obtained from corresponding standard plasma samples spiked with eleven analytes and ISs, and real plasma sample at 0.5 h after oral administration of NXTC.

#### 2.5.2. Linearity and LLOQ

The linearity was carried out by analyzing a series of calibration work solutions in duplicate over 3 consecutive days. The calibration curve was individually determined by plotting the peak area ratio of each analyte/IS (*y*) versus the nominal concentration (*x*) of each analyte. The linearity was analyzed by weighted (1/*x*^2^) least squares linear regression. The lower limit of quantification (LLOQ) defined as the signal-to-noise ratio (S/N) was higher than 5 and the relative standard deviation (RSD) was within 20%.

#### 2.5.3. Accuracy and Precision

The intraday precision and accuracy were assessed by determining LLOQ and QC samples at low, medium, and high concentration (*n* = 6) of the same day. The interday accuracy and precision were tested by performing the procedure once a day for 3 consecutive days to determine interday precision along with the standard calibration curve. The intraday and interday precisions were evaluated by using the relative standard deviations (RSDs) and the accuracy was assessed by calculating the percentage of measured concentration to the nominal concentration. Acceptance criteria of each concentration level from the nominal concentration should be less than ±15.0% and less than ±20.0% at LLOQs.

#### 2.5.4. Recovery and Matrix Effect

The recovery of 11 analytes at three concentration levels and ISs were tested by comparing the peak areas of the analytes in extracted samples with those of the postextracted spiked samples. The matrix effect of the analytes and ISs had been detected by comparing the peak areas of the analytes in postextracted spiked samples with those of corresponding standard solutions at three concentration levels. Both the recovery and matrix effect were measured in six replicates.

#### 2.5.5. Stability

The stability tests were performed to determine the analytes at room temperature for 24 h, stored at −20°C for a month and under three freeze-thaw cycles. All stability studies were performed six times at three level QC samples.

### 2.6. Determination of 11 Compounds in NXTC

The contents of paeoniflorin, ecdysterone, amygdalin, mulberroside A, caffeic acid, ferulic acid, salvianolic acid B, astragaloside IV, formononetin, cryptotanshinone, and tanshinone IIA in NXTC were quantitatively determined to calculate the administration dosage to rats. Take appropriate NXTC dissolved in methanol and ultrasonic extracted for 20 min and then centrifuged at 14,000 ×g for 10 min. 10 *μ*L of the filtrate was injected into LC-MS/MS for quantitative analysis. The contents of paeoniflorin, ecdysterone, amygdalin, mulberroside A, caffeic acid, ferulic acid, salvianolic acid B, astragaloside IV, formononetin, cryptotanshinone, and tanshinone IIA were determined as 1663.3, 45.7, 2203.3, 79.0, 14.2, 107.3, 685.3, 70.0, 78.4, 97.2, and 154.0 *μ*g/g, respectively.

### 2.7. Pharmacokinetic Study

Twenty Male Sprague-Dawley rats (220–250) g were obtained from Shan Chuanhong experimental animals Technology Co., Ltd. (Beijing, China). Rats had been bred in a breeding room (12 h dark-light cycle; temperature was 23°C ± 2°C and humidity was 50 ± 5%) and fed standard laboratory food and water for a week to acclimatization. The animal experiment was conducted according to the Institute's Guide for the Care and Use of Laboratory Animals of Tianjin University of Traditional Chinese Medicine. Rats were fasted for 12 h with free access to water before the experiment. NXTC extracts were orally administrated to ten rats at single time dose (0.5 g kg^−1^) and to ten rats at triple doses (1.5 g kg^−1^). The blood samples were collected at 5, 10, 15, 30, 45, 60, 90, 120, 240, 480, 720, and 1440 min after oral administration. Blood samples (200 *μ*L) were collected from the vein of the eye sockets into heparinized tubes after the rats were anesthetized with ethyl ether. The samples were immediately centrifuged at 7,000 rpm, 10°C for 10 min. Then the plasma samples obtained were stored at −20°C.

### 2.8. Data Analysis 

Some pharmacokinetic parameters were calculated by DAS1.0 software (Drug and Statistics 1.0, Medical College of Wannan, China). An appropriate model was employed to calculate the following parameters: area under the plasma concentration versus time curve from zero to last sampling time (AUC_0→24 h_) and infinity (AUC_0→*∞*_) and mean retention time from zero to last sampling time (MRT_0→24 h_). The maximum drug concentration in plasma (*C*_max_) and the time to reach maximum drug concentration (*T*_max_) were directly obtained from individual plasma concentration-time data observed. All data were shown as average value ± standard deviation.

## 3. Results and Discussion

### 3.1. Method Development

#### 3.1.1. Optimization of Mass Spectrometry

In order to achieve maximum responses of all analytes, the MS conditions had been optimized by infusing their standard solution to the mass spectrometer. Both positive and negative ionization modes were optimized and the full-scan mass spectra showed that the signals of cryptotanshinone, tanshinone IIA, and astragaloside IV in positive ion mode were higher than those in negative ion mode while the other analytes show higher values in negative ion mode than those in positive ion mode. Finally, the source parameters were optimized to achieve the maximum abundance of the molecular ions of the compounds. All results were listed in Table 1. Geniposide, chlorogenic acid, puerarin, and sennoside B were selected as internal standards to determine glycoside (paeoniflorin, ecdysterone, amygdalin, mulberroside A, and astragaloside IV), phenolic acids (caffeic acid, ferulic acid, and salvianolic acid B), flavonoids (formononetin), and tanshinone (cryptotanshinone and tanshinone IIA), respectively.

#### 3.1.2. Optimization of Chromatography Condition

To achieve good peak symmetry and sufficient ionization response for each analyte, a gradient mobile phase system with acetonitrile-water was optimized. It was found that ferulic acid, caffeic acid, and salvianolic acid B have good peak symmetry when 0.1% formic acid was added in the water. Thus, acetonitrile-water containing 0.1% formic acid system was selected as mobile phase.

#### 3.1.3. Optimization of Extraction Conditions

The extraction conditions were focused on decreasing the matrix effect and increasing extraction recovery. In this study, two commonly used blood sample treatment methods including liquid-liquid extraction (LLE) with ethyl acetate and protein precipitation (PPT) with acetonitrile were evaluated. It was found that the extraction recovery of LLE with ethyl acetate was very low due to the complicated polarity of the analytes in this case. The PPT with acetonitrile showed higher extraction efficiency and lower noise level. Therefore, protein precipitation (PPT) with acetonitrile was selected to prepare the samples.

### 3.2. Method Validation

#### 3.2.1. Specificity

**Figure 1:**
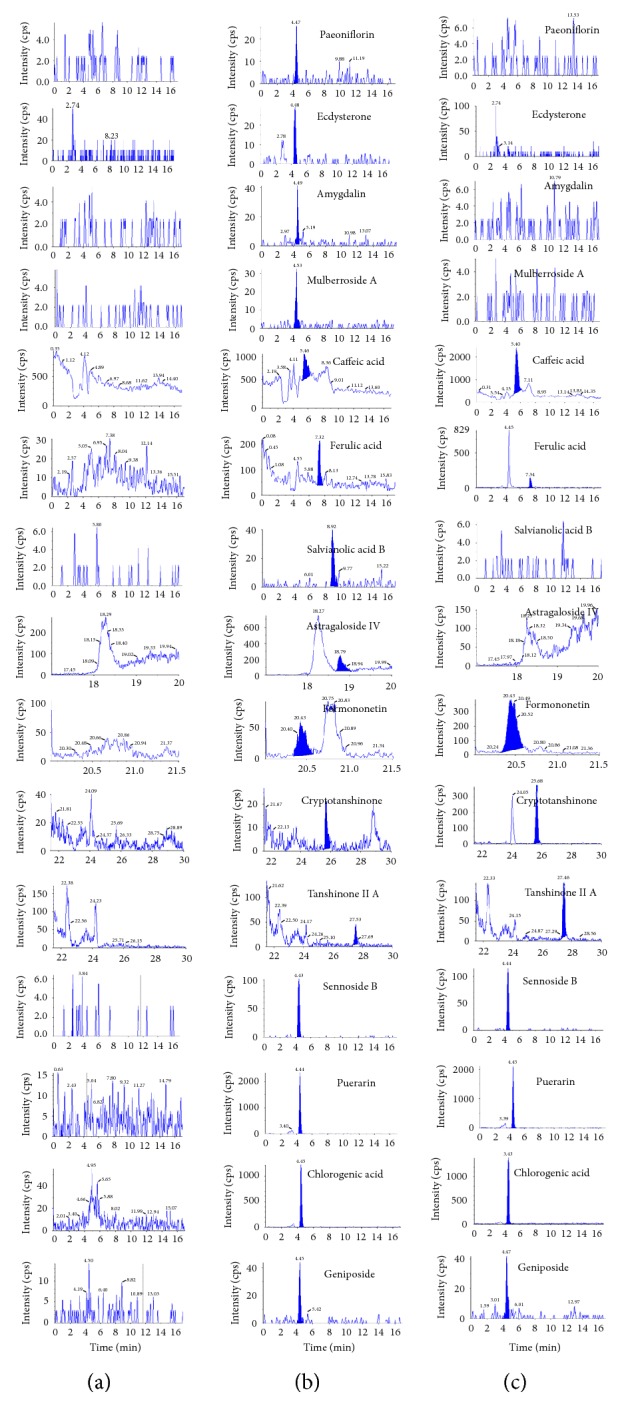
The chromatograms of the analytes in rat plasma: blank plasma (a), blank rat plasma spiked with standard compounds (b), and plasma samples taken from rats 30 min after oral administration of traditional Chinese medicinal preparation of Naoxintong capsule (c).

Typical chromatograms of blank sample, standard sample (LLOQ), and plasma samples are shown in Figure 1, respectively. The analytes were well separated and no endogenous interference was observed at retention time of paeoniflorin (4.47 min), ecdysterone (4.48 min), amygdalin (4.49 min), mulberroside A (4.53 min), caffeic acid (5.28 min), ferulic acid (min 7.32), salvianolic acid B (8.92 min), astragaloside IV (18.79 min), formononetin (20.43 min), cryptotanshinone (25.64 min), tanshinone IIA (27.53 min), and four ISs such as sennoside B (4.43 min), puerarin (4.44 min), chlorogenic acid (4.45 min), and geniposide (4.47 min) as shown in Figure 1.

#### 3.2.2. Calibration Curve and Lower Limits of Quantification

The standard calibration curves for spiked rat plasma containing paeoniflorin, ecdysterone, amygdalin, mulberroside A, caffeic acid, ferulic acid, salvianolic acid B, astragaloside IV, formononetin, cryptotanshinone, and tanshinone IIA were linear within the concentration rang of 2.5–750 ng mL^−1^, 5–1500 ng mL^−1^ 10–3000 ng mL^−1^, 3–3000 ng mL^−1^, 3–3000 ng mL^−1^, 1.5–1500 ng mL^−1^, 10–3000 ng mL^−1^, 10–3000 ng mL^−1^, 0.075–75 ng mL^−1^, 0.075–75 ng mL^−1^, and 0.3–300 ng mL^−1^ and the regression equations are as shown in Table 2. The lower limit of quantifications (LLOQs) for determination of 11 compounds in plasma were 2.5 ng mL^−1^, 5 ng mL^−1^, 10 ng mL^−1^, 3 ng mL^−1^, 3 ng mL^−1^, 1.5 ng mL^−1^, 10 ng mL^−1^, 10 ng mL^−1^, 0.075 ng mL^−1^, 0.075 ng mL^−1^ and 0.3 ng mL^−1^, respectively. The accuracy of LLOQ was from 98.3% to 105% and the relative standard deviation (RSD) (*n* = 6) was less than 14%. All data is listed in Table 2.

#### 3.2.3. Precision and Accuracy

The precision and accuracy of the method are summarized in Table 3. The assay values for both intra- and interday were found to be within the accepted variable limits. The RSD% of intra- and interday was below 15%, and the accuracy was within the range from 85 to 114%. The results demonstrated that the method was accurate and reproducible for determination of all analytes in rat plasma.

#### 3.2.4. Extraction Recovery and Matrix Effect

The extraction recoveries and matrix effects of the method are summarized in Table 4. The extraction recoveries of all analytes ranged from 60% to 107% at three-level QC samples and their RSDs were less than 15%. The matrix effects of all analytes except for paeoniflorin ranged from 85.2% to 115% for three-level QC samples and their RSDs were less than 15%. Although the matrix effects of paeoniflorin were from 127% to 152%, its RSDs were less than 15%. It was concluded that protein precipitation with acetonitrile proved to be precise and could be used to extract the analytes from the plasma sample.

#### 3.2.5. Stability

The stability of all the analytes under various conditions is presented in Table 5. It was found that the these analytes were stable in rat plasma after room temperature for 24 h, stored at −20°C for one month, under three freeze-thaw cycles. The above results demonstrate that the developed HPLC-MS/MS method could be used to determine all these analytes in rat plasma.

### 3.3. Assaying the Dosage of Oral Administration of 11 Components

The contents of 11 components in NXTC capsule were determined by the HPLC-MS/MS method. The results showed that the oral dose of paeoniflorin, ecdysterone, amygdalin, mulberroside A, caffeic acid, ferulic acid, salvianolic acid B, astragaloside IV, formononetin, cryptotanshinone, and tanshinone IIA was 831.7 *μ*g kg^−1^, 22.9 *μ*g kg^−1^, 1101.7 *μ*g kg^−1^, 39.5 *μ*g kg^−1^, 7.1 *μ*g kg^−1^, 53.7 *μ*g kg^−1^, 342.7 *μ*g kg^−1^, 35.0 *μ*g kg^−1^, 39.2 *μ*g kg^−1^, 48.6 *μ*g kg^−1^, and 77 *μ*g kg^−1^ after oral administration of NXTC at doses of 0.5 g kg^−1^, respectively.

### 3.4. Pharmacokinetic Study

**Figure 2:**
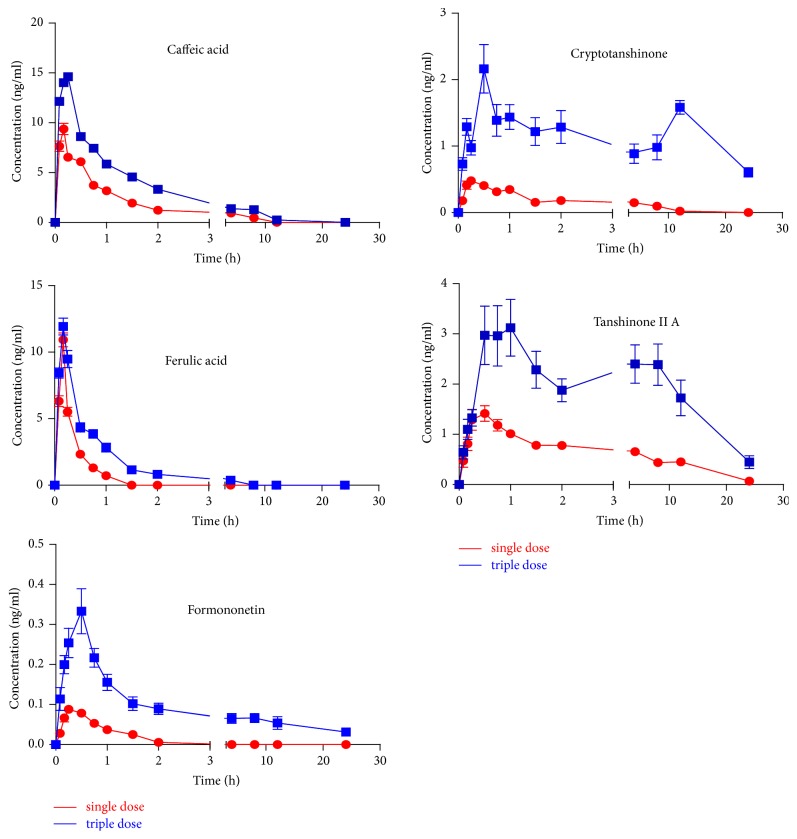
The mean plasma concentration-time profiles of caffeic acid, ferulic acid, formononetin, cryptotanshinone, and tanshinone IIA after oral administration of Naoxintong capsule at doses of 0.5 g kg^−1^ and 1.5 g kg^−1^ (*n* = 10).

The developed HPLC-MS/MS method was successfully used to determine the plasma concentrations of 11 components after oral administration of NXTC at doses of 0.5 g kg^−1^ and 1.5 g kg^−1^, respectively. The results showed that 5 components (caffeic acid, ferulic acid, formononetin, cryptotanshinone, and tanshinone IIA) could be detected in rat plasma after oral administration of NXTC while the other 6 components (paeoniflorin, ecdysterone, amygdalin, mulberroside A, salvianolic acid B, and astragaloside IV) were not detected at the present LLOQ level. The result indicated that some more sensitive methods would be established and validated to focus on the mechanism of absorption and metabolism of these six components. However, the LLOQs of the new method were sufficient to characterize the pharmacokinetics of the other 5 components (caffeic acid, ferulic acid, formononetin, cryptotanshinone, and tanshinone IIA) in rats. The partial main pharmacokinetic parameters of caffeic acid, ferulic acid, formononetin, cryptotanshinone, and tanshinone IIA in rats are shown in Table 6. The mean plasma concentration-time profiles of caffeic acid, ferulic acid, formononetin, cryptotanshinone, and tanshinone IIA are shown in Figure 2. It was observed that *T*_max_ of 3 components (caffeic acid, ferulic acid, and formononetin) were less than 1 h, which implied that they were rapidly absorbed in rat plasma after oral administration of NXTC at doses of 0.5 g kg^−1^ and 1.5 g kg^−1^. Ferulic acid, as a hydroxycinnamic acid, is a well-known antioxidant compound and has the cardiovascular protection function [19]. It is used for treating age-related diseases such as neurodegenerative disorders and cardiovascular disease [20]. The pharmacokinetic results indicated that the mean *T*_max_ after single dose administration of NXT capsule was 0.17 h. It was six times higher than the mean *T*_max_ of 0.03 h after a single oral administration of ferulic acid [21], which implies other components in NXTC might affect the absorption of ferulic acid. All *C*_max_ and AUC_(0–24 h)_ values of 5 components (caffeic acid, ferulic acid, formononetin, cryptotanshinone, and tanshinone IIA) after oral administration of NXT capsule at triple doses of 1.5 g kg^−1^ were higher than those after oral administration at normal doses of 0.5 g kg^−1^. It was indicated that the absorptions of these 5 components in vivo were promoted with the increase of the oral dose of administration of NXTC. Cryptotanshinone and tanshinone IIA have attracted particular attention from medicinal chemists and clinicians due to their diverse biological activities, such as antihypertensive effect and antithrombus and antiplatelet aggregation activities [22, 23].* T*_1/2_ values of cryptotanshinone (17.8 ± 17.1 h) and tanshinone IIA (17.8 ± 17.1 h) indicated that the action time of both cryptotanshinone and tanshinone IIA were significantly longer in body than the other three components. As shown in Figure 2, two plasma concentration peaks were observed in the mean plasma concentration curves profiles of cryptotanshinone and tanshinone IIA, which was consistent with results from previous studies [24]. Caffeic acid has very excellent antioxidant and antiviral activity [25]. From Table 6, it was found that values of *T*_max_ and* T*_1/2_ of caffeic acid and ferulic acid were much closer, demonstrating that they have similar absorption and elimination rate. The *C*_max_ values were 15.24 ± 3.08 ng mL^−1^ and 12.07 ± 4.61 ng mL^−1^ and values of AUC_(0–24 h)_ were 25.99 ± 18.62 ng mL^-1 ^h^−1^and 8.33 ± 3.87 ng mL^−1^ h^−1^ for caffeic acid and ferulic acid at triple doses, respectively. The results were not correspondent with their dose administered to the rats (7.1 *μ*g kg^−1^ caffeic acid and 53.7 *μ*g kg^−1^ ferulic acid). The possible reason may be that some of the caffeic acid detected in rat plasma was the metabolites of the components in NXTC. It was reported that the medicinal herbs containing formononetin often have been used to treat cardiovascular diseases for a long time [26]. In the present studies, *C*_max_ of formononetin was less than 1 ng mL^−1^. The AUC_(0–24 h)_ values were lower than 1 ng mL^−1^. These showed that formononetin might still be a mainly active ingredient that we can detect in rat plasma. Based on the above results, the newly established LC-MS/MS method was sufficiently sensitive for the determination of multiple components in rat plasma and was suitable to simultaneously evaluate the pharmacokinetic properties of the multiple bioactive components after oral administration of NXTC.

## 4. Conclusion

A rapid, reliable, and sensitive HPLC-MS/MS method was validated and optimized for quantitative analysis of paeoniflorin, ecdysterone, amygdalin, mulberroside A, caffeic acid, ferulic acid, salvianolic acid B, astragaloside IV, formononetin, cryptotanshinone, and tanshinone IIA in rat plasma. To our knowledge, this is the first time to evaluate the pharmacokinetics of NXTC after oral administration. It could be useful to the clinical application and for the quality control of the medicine. The pharmacokinetic parameters obtained from this study and the validated method would be useful in clinical applications of TCM preparations of Naoxintong capsule.

## Figures and Tables

**Table 1:** Source and MRM parameters of the eleven compounds and four ISs.

Compounds	Parameter											
	CUR	CAD	IS	TEM	GS1	GS2	*Q*1	*Q*3	DP(V)	EP(V)	CE(V)	CXP(V)
Paeoniflorin	20	8	−4500	500	30	30	478.6	121.1	−45	−4	−30	−1
Ecdysterone	20	8	−4500	500	30	30	478.9	159.0	−60	−10	−37	−1
Amygdalin	20	8	−4500	500	30	30	455.8	322.8	−50	−4.5	−20	−22
Mulberroside A	20	8	−4500	500	30	30	567.0	242.6	−60	−10	−38	−16
Caffeic acid	20	8	−4500	500	30	30	178.9	134.9	−35	−3.5	−24	−0.5
Ferulic acid	20	8	−4500	500	30	30	192.8	133.9	−31	−4	−23	−1
Salvianolic acid B	20	8	−4500	500	30	30	716.5	518.5	−55	−4.5	−25	−40
astragaloside IV	10	8	5000	500	40	30	807.0	807.0	140	10	50	55
Formononetin	20	8	−4500	500	30	30	267.0	251.8	−60	−2	−27	−16
Cryptotanshinone	10	8	5000	500	40	30	297.0	254.2	50	9	35	15
Tanshinone IIA	10	8	5000	500	40	30	295.2	277.1	58	10	27	21
Geniposide	20	8	−4500	500	30	30	387.0	224.7	−40	−9	−15	−15
Sennoside B	20	8	−4500	500	30	30	860.6	385.9	−87	−10	−60	−2
Puerarin	20	8	−4500	500	30	30	414.8	266.9	−50	−9	−46	−2
Chlorogenic acid	20	8	−4500	500	30	30	352.8	190.9	−33	−4	−32	−2

**Table 2:** Linear regression data, LLODs, and accuracy for the eleven compounds.

Compounds	Regression equation	*r*	Linearity range (ng*∗*mL^−1^)	LLOD (ng*∗*mL^−1^)	Accuracy (%)	RSD (%)
Paeoniflorin	*Y* = 0.0124*x* + 0.0522	0.9997	2.5–750	2.5	99.5	13
Ecdysterone	*Y* = 0.0128*x* − 0.000782	0.9985	5–1500	5	100	6.3
Amygdalin	*Y* = 0.016*x* + 0.1114	0.9976	10–3000	10	99.2	12
Mulberroside A	*Y* = 0.0217*x* − 0.0373	0.9910	3–3000	3	92.3	14
Caffeic acid	*Y* = 0.083*x* + 1.12	0.9993	3–3000	3	100	7.4
Ferulic acid	*Y* = 0.00894*x* + 0.0224	0.9962	1.5–1500	1.5	91.4	5.4
Salvianolic acid B	*Y* = 0.00541*x* − 0.0233	0.9972	10–3000	10	105	7.8
Astragaloside IV	*Y* = 0.3*x* − 2.28	0.9982	10–3000	10	99.7	6.9
Formononetin	*Y* = 0.00216*x* + 0.00183	0.9958	0.075–75	0.075	99.2	4.4
Cryptotanshinone	*Y* = 0.0589*x* − 0.28	0.9916	0.075–75	0.075	98.3	4.4
Tanshinone IIA	*Y* = 0.0387*x* − 0.0776	0.9975	0.3–300	0.3	101	3.5

**Table 3:** Intraday and interday accuracy and precision of eleven compounds (*n* = 6).

Compounds	Concentration (ng*∗*mL^−1^)	Intraday		Interday	
		Accuracy (%)	RSD (%)	Accuracy (%)	RSD (%)
Paeoniflorin	7.5	93.0	14	95.7	2.5
	75	101	8.7	102	3.8
	750	96.6	6.3	101	1.2
Ecdysterone	15	111	8.4	102	2.5
	150	106	12	99.6	3.0
	1500	101	6.7	97.7	5.2
Amygdalin	30	98	10	101	1.9
	300	100	11	99.1	3.5
	3000	102	11	101	0.8
Mulberroside A	30	88	13	96.6	2.4
	300	104	12	90.9	8.3
	3000	114	3	98.8	12
Caffeic acid	30	103	8.5	102	3.0
	300	85	9.4	95.2	10
	3000	103	7.8	95.0	4.2
Ferulic acid	15	106	10	98.0	10
	150	111	7.7	102	7.0
	1500	113	2.9	106	4.5
Salvianolic acid B	30	103	10	97.2	9.4
	300	109	6	102	4.1
	3000	100	12	106	9.0
Astragaloside IV	30	90.3	14	94.7	4.9
	300	107	10	102	8.2
	3000	103	8.4	101	4.8
Formononetin	0.75	107	3.4	100	1.1
	7.5	105	14	101	8.2
	75	114	7.3	101	1.7
Cryptotanshinone	3	90	9.4	97.9	3.2
	30	97.4	14	101	13
	300	103	11	103	5.5
Tanshinone IIA	0.75	104	6.1	100	2.4
	7.5	101	15	98.7	5.8
	75	104	13	97.5	4.4

**Table 4:** Recoveries and matrix effects of eleven compounds (*n* = 6).

Compounds	Concentration (ng*∗*mL^−1^)	Recovery		Matrix effect	
		Mean (%)	RSD (%)	Mean (%)	RSD (%)
Paeoniflorin	7.5	100	7.8	152	12
	75	96.6	12	127	11
	750	83.6	12	146	12
Ecdysterone	15	98.9	6.2	80.0	12
	150	83.6	9.6	95.8	11
	1500	104	7.0	110	13
Amygdalin	30	107	12	78.0	4.8
	300	90.5	7.8	82.6	7.8
	3000	87.2	13	75.7	11
Mulberroside A	30	78.7	4.9	84.3	5.6
	300	92.0	15	76.0	14
	3000	76.6	5.9	73.7	8.2
Caffeic acid	30	98.2	7.1	96.4	3.6
	300	81.8	12	107	9.9
	3000	88.7	6.3	109	7.5
Ferulic acid	15	91.6	6.5	101	3.7
	150	79.6	6.8	89.5	4.4
	1500	93.2	7.5	92.6	8.9
Salvianolic acid B	30	82.6	14	105	11
	300	62.7	10	82.7	6.2
	3000	81.2	8.5	76.0	9.7
Astragaloside IV	30	91.0	9.0	109	9.3
	300	95.2	4.7	109	14
	3000	97.1	13	115	3.0
Formononetin	0.75	87.9	7.5	98.5	4.2
	7.5	103	12	87.7	9.3
	75	99.7	13	106	13
Cryptotanshinone	3	97.0	14	75.3	12
	30	76.2	8.2	89.0	4.6
	300	76.0	14	79.0	5.5
Tanshinone IIA	0.75	99.6	9.3	92.0	5.2
	7.5	60.0	14	96.0	8.4
	75	81.5	6.6	90.0	11

**Table 5:** 24 h storage at room temperature stability, three freeze-thaw cycles stability and long-term stability of eleven compounds (*n* = 6).

Compound	Concentration (ng mL^−1^)	Freeze-thaw cycles		At −20°C for 1 weeks		Autosampler for 24 h	
		Remains (%)	RSD (%)	Remains (%)	RSD (%)	Remains (%)	RSD (%)
Paeoniflorin	7.5	93.7	14	101	8.8	95.8	15
	75	88.0	8.6	85.8	9.5	97.4	6.4
	750	106	5.0	96.5	13	89.9	11
Ecdysterone	15	97.1	11	102	11	94.2	8.4
	150	95.5	14	107	11	102	7.0
	1500	95.0	8.0	103	12	93.1	6.2
Amygdalin	30	87.0	10	93.0	7.2	98.1	9.0
	300	87.9	12	105	14	94.2	15
	3000	103	10	94.1	13	92.6	12
Mulberroside A	30	104	11	112	12	86.6	10
	300	94.4	14	106	5.7	87.7	9.9
	3000	96.5	9.9	97.2	13	95.3	9.3
Caffeic acid	30	106	8.5	95.8	13	103	8.9
	300	85.6	14	101	11	89.9	12
	3000	89.2	3.5	90.5	11	85.2	7.7
Ferulic acid	15	111	11	105	9.7	107	12
	150	108	11	111	11	93.2	8.7
	1500	99.6	4.1	106	7.5	95.0	7.7
Salvianolic acid B	30	97.1	11	113	13	91.7	6.4
	300	105	8.8	92.6	9.4	99.9	12
	3000	101	12	100	13	108	8.5
Astragaloside IV	30	111	4.7	113	6.7	86.3	10
	300	103	4.1	108	8.8	101	12
	3000	95.4	10	97.4	14	96.1	14
Formononetin	0.75	94.5	6.2	111	8.6	102	13
	7.5	89.9	12	88.9	6.6	111	12
	75	90.0	9.0	106	1.8	108	8.1
Cryptotanshinone	3	104	12	112	8.7	87.2	13
	30	106	10	93.2	8.5	85.2	12
	300	107	9.0	109	11	87.5	12
Tanshinone IIA	0.75	99.0	4.9	93.0	10	88.4	12
	7.5	114	9.1	105	14	94.9	9.0
	75	106	10	94.1	15	93.6	11

**Table 6:** Pharmacokinetic parameters of five compounds in rat plasma after oral administration of single dose and triple dose of NXT capsule. (*n* = 10, mean ± SD).

Analytes	Caffeic acid		Ferulic acid		Formononetin		Cryptotanshinone		Tanshinone IIA	
	0.5 g kg^−1^	1.5 g kg^−1^	0.5 g kg^−1^	1.5 g kg^−1^	0.5 g kg^−1^	1.5 g kg^−1^	0.5 g kg^−1^	1.5 g kg^−1^	0.5 g kg^−1^	1.5 g kg^−1^
*T*_max_ (h)	0.18 ± 0.03	0.20 ± 0.04	0.17 ± 0.00	0.19 ± 0.04	0.49 ± 0.33	0.38 ± 0.25	0.51 ± 0.40	0.60 ± 0.20	0.54 ± 0.29	3.17 ± 3.75
*C*_max_ (ng/mL)	9.80 ± 4.40	15.24 ± 3.08	10.93 ± 4.67	12.07 ± 4.61	0.11 ± 0.05	0.37 ± 0.32	0.61 ± 0.23	2.78 ± 2.52	1.61 ± 1.11	4.18 ± 3.34
*T*_1/2_ (h)	0.83 ± 0.33	0.70 ± 0.35	0.20 ± 0.19	0.42 ± 0.15	2.96 ± 5.61	1.42 ± 0.69	2.93 ± 3.89	17.8 ± 17.1	11.4 ± 15.8	13.2 ± 10.4
AUC_(0–24 h)_ (ng/mL*∗*h)	11.68 ± 9.60	25.99 ± 18.62	3.20 ± 1.55	8.33 ± 3.87	0.08 ± 0.05	1.47 ± 1.82	1.49 ± 0.76	24.8 ± 27.9	8.15 ± 4.85	35.4 ± 42.2
AUC_(0–*∞*)_ (ng/mL*∗*h)	19.57 ± 21.12	28.52 ± 20.01	4.19 ± 2.30	14.4 ± 12.8	0.15 ± 0.09	3.06 ± 3.07	4.72 ± 5.99	55.2 ± 59.8	27.7 ± 36.2	83.0 ± 81.0
MRT_(0–24 h)_ (h)	1.33 ± 1.19	2.03 ± 1.43	0.28 ± 0.10	0.81 ± 0.38	0.56 ± 0.23	5.16 ± 3.68	3.06 ± 1.47	7.37 ± 3.95	5.48 ± 1.66	5.75 ± 3.36
MRT_(0–*∞*)_ (h)	4.02 ± 4.96	2.72 ± 1.82	0.48 ± 0.33	3.80 ± 6.11	1.67 ± 1.53	25.9 ± 31.8	20.7 ± 27.7	19.0 ± 17.5	26.8 ± 31.5	27.2 ± 20.6
